# Strategies for Generating Diverse Antibody Repertoires Using Transgenic Animals Expressing Human Antibodies

**DOI:** 10.3389/fimmu.2018.00460

**Published:** 2018-03-07

**Authors:** Weihsu C. Chen, Christopher M. Murawsky

**Affiliations:** ^1^Biologics Discovery, Department of Therapeutic Discovery, Amgen British Columbia Inc., Burnaby, BC, Canada

**Keywords:** antibody, repertoire, transgenic, therapeutic, engineering, B cell, diversity, immune

## Abstract

Therapeutic molecules derived from antibodies have become a dominant class of drugs used to treat human disease. Increasingly, therapeutic antibodies are discovered using transgenic animal systems that have been engineered to express human antibodies. While the engineering details differ, these platforms share the ability to raise an immune response that is comprised of antibodies with fully human idiotypes. Although the predominant transgenic host species has been mouse, the genomes of rats, rabbits, chickens, and cows have also been modified to express human antibodies. The creation of transgenic animal platforms expressing human antibody repertoires has revolutionized therapeutic antibody drug discovery. The observation that the immune systems of these animals are able to recognize and respond to a wide range of therapeutically relevant human targets has led to a surge in antibody-derived drugs in current development. While the clinical success of fully human monoclonal antibodies derived from transgenic animals is well established, recent trends have seen increasingly stringent functional design goals and a shift in difficulty as the industry attempts to tackle the next generation of disease-associated targets. These challenges have been met with a number of novel approaches focused on the generation of large, high-quality, and diverse antibody repertoires. In this perspective, we describe some of the strategies and considerations we use for manipulating the immune systems of transgenic animal platforms (such as XenoMouse^®^) with a focus on maximizing the diversity of the primary response and steering the ensuing antibody repertoire toward a desired outcome.

## Introduction

The remarkable capacity of engineered animals to utilize human antibody sequences to functionally replace their own has allowed researchers to harness the power of the natural humoral immune response. Antibody generation *in vivo* offers several advantages, including the ability to readily recover molecules that bind to the target antigen with high specificity and affinity (1). The processes of *in vivo* sequence diversification, antigen-driven somatic hypermutation, and numerous quality control checkpoints ensure the non-random selection and enrichment of B cells that produce antibodies with therapeutically desirable properties (2–5).

Despite the demonstrated successes of transgenic platforms, researchers face significant challenges related to the increasing complexity of functional design goals and targets. Ideal antibody candidates are often required to bind with high affinity to a specific epitope, cross-react to a non-human ortholog, lack binding to paralogs, and survive the rigors of the stringent drug development process (6). Thus, antibodies satisfying all the design goals may be extremely rare, if they are elicited at all. At the same time, the targets themselves have shifted in difficulty from the “low-hanging fruit” to those that are considerably more challenging (7–11). In this perspective, we will highlight the strategies and considerations used for manipulating the immune systems of transgenic animal platforms. We will first concentrate on the transgenic platforms and call out specific features that contribute to forming antibody repertoires. We will then draw from our experience using XenoMouse^®^ to discover novel human therapeutics and focus on the approaches we use to maximizing the diversity of the primary antibody repertoire and to steer it toward the desired outcome.

## Transgenic Platforms Expressing Human Antibody Repertoires

The collection of unique B cells in an organism (the B-cell repertoire) encodes and produces the corresponding antibody repertoire. Herein, we will use “antibody repertoire” to describe the collection of sequence-unique antibodies, and their corresponding B cells, present in a given system. Transgenic animal platforms expressing human antibodies utilize the *in vivo* biology of the host immune system to generate diversity through canonical recombination and somatic hypermutation. Importantly, both the breadth and shape of the antibody repertoire can be influenced to yield a desired response.

The demonstration that large portions of the intact human immunoglobulin loci could be introduced into the mouse genome was a significant achievement and is the subject of a series of excellent reviews (1, 12–14). These efforts culminated in the world’s first fully human transgenic antibody generation platforms (XenoMouse^®^ and HuMab-Mouse^®^) and have been followed by a series of related, next-generation animals (15–20). These platforms largely recapitulate critical aspects of the human antibody repertoire including V-, D-, and J-segment usage patterns. The remarkable ability of these animals to assimilate mouse biology and human antibody sequence information has revolutionized biotechnology by providing access to a diverse source of fully human antibodies. The number of marketed human therapeutics derived from these platforms, and their continued use as engines for *de novo* antibody discovery, highlights their success (21).

Three exciting, emerging trends in this area are as follows: (1) the development of human antibody generation platforms in species other than mouse, (2) the creation of transgenic systems that produce non-canonical antibodies, and (3) attempts to genetically manipulate the immune system to yield unconventional antibody repertoires.

Open Monoclonal Technologies (OMT, now Ligand Pharmaceuticals) produced a set of transgenic rat strains (OmniRat^®^) that express a human idiotype repertoire (22, 23). Crystal Biosciences (now Ligand Pharmaceuticals) and Sab Biotherapeutics have reported the development of transgenic chickens (OmniChicken™) and cows (Tc Bovine™), respectively, carrying human repertoires (24–27). The ability to produce human antibody repertoires in evolutionarily divergent systems may be advantageous for helping overcome tolerance. The immune systems of these animals are distinct from conventional antibody generation hosts. As such, they are potentially rich sources of antibody repertoires that may help address challenging targets or design goals.

Non-canonical antibodies have the potential to address practical drug development challenges and to bind to targets in novel ways. The desire to create multispecific therapeutics, with human idiotypes at their core, has seen the development of platforms designed to yield candidates suitable for straightforward conversion into multivalent formats (e.g., common light chain animals) (28). However, the most interesting molecules of this class are derived from heavy-chain-only antibodies (HCAbs) (29, 30). Because of their small size and unique mechanism by which they interact with antigens, the VH domains derived from these antibodies have the potential to bind to classes of targets that regular antibodies cannot. Several groups have developed transgenic HCAb generation platforms that utilize a human antibody repertoire (Harbour Biomed, OMT Therapeutics, Crescendo) (31, 32).

Additional transgenic modifications, distinct from the immunoglobulin loci, can impact the resulting antibody repertoire. Researchers at ImmunoGenes have produced a mouse that over expresses the bovine neonatal Fc receptor (FcRn) (33–35). These animals have an enhanced humoral response and an expanded IgM repertoire (36). Editing the genome by manipulating genes that are known to impact the humoral response would be expected to allow researchers to modify the resulting repertoire. Combining these modifications with a human antibody discovery platform may yield animals capable of producing nonstandard repertoires.

## Strategies for Maximizing Antibody Repertoire Diversity

The primary goal for our XenoMouse^®^ immunization campaigns is to maximize antibody repertoire diversity. This serves two purposes: (1) it increases the likelihood of finding antibodies that meet the functional design goals and (2) it increases the probability of identifying lead candidates that are sequence diverse (6). While identification of an antibody satisfying the design goals is a minimal requirement for a successful campaign, the stringent manufacturing criteria of therapeutic antibody development also necessitate the selection of multiple lead candidates with different sequences. If the initial candidate fails biophysical characterization, a unique backup molecule has already been identified.

Since the number of B cells in a mouse at any given moment is ~10^8^, and the theoretical diversity of the human antibody repertoire exceeds 10^11^, it is reasonable to conclude that an individual mouse can only harbor a fraction of the potential human repertoire (37, 38). As such, a fundamental tenet of our strategy is that individual mice can produce unique antibody repertoires. We regularly immunize large cohorts of mice to maximize exploration and sampling of the theoretical antibody sequence space. In addition, we have developed multiple strains of XenoMouse^®^ for the purpose of generating the broadest repertoire possible (39). Indeed, we routinely observe biases among the strains with regard to their response to specific antigens. To ensure we raise a diverse response, we include many animals from a variety of strains and genetic backgrounds in our antibody discovery campaigns.

A second guiding principal that governs our approach for maximizing the diversity of antibody repertoires is to develop a comprehensive immunization strategy. Multiple factors should be considered when designing a strategy focused on generating a broad antibody response. These include (1) preparation and presentation of the immunogen, (2) choice of adjuvant, (3) immunization method, and (4) the impact of T-cell tolerance. Although the precise details depend on the nature of the target antigen, our general approach is to use validated forms of immunogens and present them to the immune system in variety of ways.

Preparing the target antigen such that it is an effective immunogen is a critical first step. When considering ways to prepare immunogens, we focus on strategies that are the least likely to disrupt the native structure of the antigen and destroy important epitopes. Our goal is to produce at least three orthogonal types of immunogens for each target: soluble protein, cells expressing the target on the membrane, and DNA expression constructs. These immunogens form the core of our primary immunization strategy and allow us to present multiple forms of the antigen to the immune system.

Many target antigens can be engineered to yield soluble proteins for use as immunogens. The extracellular domains (ECDs) of type-I and -II membrane proteins often exist as modular domains that can be isolated from their native context without compromising their structure. For membrane targets that contain ECDs that are difficult to purify or that cannot easily be overexpressed, an alternative strategy is to artificially tether them to the surface of a cellular expression host (40). This can be done by fusing the ECD to another well-characterized transmembrane domain or by adding a signal sequence for a C-terminal Glycosylphosphatidylinositol (GPI)-anchor (41, 42). Multipass membrane proteins that contain extracellular loops (ECLs) present significant challenges for antigen engineering. Attempts to utilize the isolated loop sequences as peptide immunogens are rarely successful, presumably because they lack native conformation (43). Because of this, we favor genetic and cell-based immunogens for this target class, both of which can be readily produced using the same expression plasmid (44, 45). Regardless of how the antigen is engineered, it is important to validate it prior to use. Evidence of the native conformation can be obtained using functional bioassays or by assessing relevant biophysical interactions.

Adjuvants play a pivotal role in potentiating immune responses against antigens. The most widely used adjuvants include the water-in-oil emulsions and the aluminum salts (46). Modern adjuvant research has focused on exploiting the connection between the innate and adaptive immune systems. In particular, toll-like receptor (TLR) agonists have received significant attention because of the important role of TLRs in the activation of innate signaling pathways (47, 48). In our experience, adjuvants are absolutely required for generating a robust immune response and diverse antibody repertoires in XenoMouse^®^. Consistent with our results, several studies have demonstrated a broadening of the antibody repertoire as a result of preparing immunogens with specific adjuvants (49, 50). Our adjuvant strategy is to leverage the most potent immunomodulatory agents available, while minimizing undesirable effects on the antigen and animal welfare. Although progress continues to be made to develop new adjuvants, we will not cover this here and direct the reader to several excellent reviews (51–54).

A successful immunization campaign requires a suitable immunogen delivery method. Both the form of the antigen and the route by which it is delivered are known to impact the resulting immune response (55–62). While we leverage many traditional methods for delivering protein and cell-based immunogens, we have had tremendous success immunizing XenoMouse^®^ using a genetic approach. Genetic immunization offers many advantages compared with traditional methods. Minimally, it requires only plasmid DNA encoding the antigen of interest which, upon delivery, is taken up by cells and expressed *in vivo*. The simplicity and native-like expression of this method increases the probability that the natural conformation of the antigen is maintained. This strategy is particularly attractive for complex membrane proteins (e.g., GPCRs, transporters, and ion channels) due to difficulties associated with their expression and purification. We have explored different approaches to improve the effectiveness of DNA immunization. These include frequent and extended boosting regimens, the use of more effective delivery methods, and inclusion of molecular adjuvants. For example, intradermal DNA delivery *via* a gene gun is a widely used approach that relies on the transfer of plasmid DNA-coated gold particles into the skin *via* particle bombardment (63, 64). The immune response can be potentiated by the inclusion of molecular adjuvants (e.g., cytokines, transcription factors, TLR agonists, etc.) (65–68). Using this method, we routinely generate immune animals within 4–5 weeks that harbor diverse antibody repertoires directed against therapeutically interesting complex membrane targets.

The ability to overcome immune tolerance remains a central problem for antibody discovery using transgenic platforms. For our therapeutic antibody campaigns using XenoMouse^®^, we have exploited observations that tolerance can often be overcome by adding T-cell epitopes (TCEs) to antigens. Consistent with our results, several groups have raised functionally active antibodies against murine antigens in wild-type mouse hosts using this strategy (69, 70). The context in which the TCE resides within the antigen plays an important role in determining whether it is efficacious. Intracellular antigen processing, which ultimately yields the peptides for MHC class-II loading and presentation to T cells, may destroy the TCE if it is presented in a context optimal for proteolytic processing (71, 72). Thus, finding the ideal location within the antigen of interest is a critical factor that must be determined empirically.

A number of inbred mouse strains have been identified that have tolerance defects that result in auto-antibody production (73). Breeding a human antibody transgenic mouse to such a strain theoretically produces an animal more prone to break tolerance. A more direct approach is to abolish expression of the murine ortholog pertaining to the human target antigen of interest (74). Indeed, we have developed a collection of XenoMouse^®^ knockout lines that carry inactivating mutations in a variety of genes orthologous to human therapeutic targets. However, major caveats are associated with these strategies: (1) many of the auto-immune strains suffer from health issues, (2) some genes are essential for embryonic development and cannot be systemically knocked out, and (3) inactivating genes required for mounting an immune response compromises the ability of the platform to function as desired. Nonetheless, in cases where it is possible, this approach is an effective tolerance-breaking strategy.

## Strategies for Steering Antibody Repertoires

The ability to “steer” the immune response toward a desired outcome has been recognized since Jenner’s revolutionary demonstration of induced immunity to the smallpox virus in the 1770s (75). Since then, multiple repertoire engineering approaches have been used with varying degrees of success. In the context of therapeutic antibody development, there is often a desire to target (or avoid) a particular epitope on the antigen of interest. Here, we will focus on a selection of current strategies that we use to direct the XenoMouse^®^ immune system to produce a desired antibody repertoire.

One of the most powerful approaches for directing the antibody repertoire toward a specific epitope is to engineer the antigen such that the desired region is effectively presented to the immune system. One way to accomplish this is to selectively express or display the minimal domain carrying the epitope(s) of interest. Importantly, this method also allows us to avoid immune-dominant epitopes that otherwise skew the antibody repertoire toward undesired specificities (76–78). We have also had success using human-mouse chimeras and grafting the ECLs of multipass membrane proteins onto related proteins that share a common structure with the target of interest. Grafting the target loops onto a scaffold that retains key intramolecular interactions raises the likelihood that the correct conformational epitopes are retained. Moreover, it is often possible to find a suitable scaffold that has been demonstrated to have more optimal expression characteristics, better cell surface retention properties, or that can tolerate heterologous sequence fusions. This approach is particularly attractive for novel targets where no structural information exists. We have had limited success extracting ECLs and immunizing them as peptides or when grafting them onto heterologous carrier proteins, despite the existence of successful reports (79, 80). The success of this strategy depends on the degree to which the design goals are tied to a specific functional epitope or if targeting multiple epitopes within a structural region is permitted.

The safety of therapeutic antibodies must be evaluated in a preclinical setting prior to human trials. This important step of drug development usually necessitates that the clinical candidate cross-reacts to the appropriate preclinical animal species chosen for toxicology (81, 82). To ensure our lead candidates can meet this stringent design goal, we frequently incorporate the orthologous antigen from the toxicology species into our immunization protocols. One strategy is to design an immunization campaign that alternates between the human and orthologous antigen to steer the antibody repertoire toward functional antibodies that cross-react to both. Monitoring cross-reactivity throughout the course of the immunization campaign allows further customization through extended or alternative boosting protocols. In our hands, the success of this strategy is tightly correlated with the conservation of functional epitopes and becomes significantly more challenging as evolutionary distances increase.

## Concluding Remarks and Future Perspectives

The convergence of several key technologies, along with our increasing understanding of molecular and cellular immunology, is allowing researchers to purposefully manipulate the immune systems of transgenic animal platforms. Combined with the ability of these systems to produce human antibody repertoires directed against disease-associated target antigens, we are armed with profoundly powerful tools for the discovery of novel therapeutics. Advances in the fields of genetic engineering and immunization have enabled diverse antibody responses to be elicited against a variety of difficult targets. Progress in antigen engineering and immunomodulation have allowed researchers to steer the immune response toward the production of more relevant antibody repertoires.

Despite impressive progress, it is clear that transgenic animal platforms have more to offer. Although we can elicit broad and diverse repertoires, we lack suitable methods to deeply interrogate them. This is particularly problematic when seeking a B cell that produces an antibody with a rare specificity. Most current strategies rely heavily on hybridoma-driven repertoire sampling, a technique known to be notoriously biased and inefficient (83). Multiple variations on direct, single B-cell screening are also used to identify antibodies of interest (84–90). Unfortunately, these methods are generally low throughput, labor intensive, and restricted to simple assays, which limits their usefulness to mine significant fractions of the elicited repertoire. Other methods to display and screen antibody repertoires were developed, in part, to address some of these limitations (e.g., phage display). However, these approaches suffer from the fact that the native pairings are lost, making it even more difficult to recover rare antibodies (91–95).

However, notable advancements in high-throughput repertoire analysis and recovery have recently been made. It is now possible to use next-generation sequencing (NGS) to determine all of the individual sequences that make up the complete antibody repertoire of an animal (96, 97). For the first time, researchers can determine the holistic effects of their attempts to influence the repertoire (98). This will clearly impact future attempts to maximize diversity and shape repertoires by giving us unprecedented insight into the consequences of experimentation. Similarly, improvements in high-throughput techniques for single-cell isolation and screening are allowing scientists to apply these techniques to antibody discovery (99–104). Coupled with the ability to determine the corresponding antibody sequences using NGS, deep interrogation of antibody repertoires is within reach. These advancements will no doubt allow the recovery of rare antibodies with properties satisfying the demanding design goals of the next generation of therapeutic targets.

## Author Contributions

WC and CM both contributed to writing the manuscript.

## Conflict of Interest Statement

WC and CM are full-time employees of, and hold stock in, Amgen, Inc.
